# Antenatal Prediction of Shoulder Dystocia and Birth Trauma Using Routine Maternal and Ultrasound Variables: Retrospective Cohort Study

**DOI:** 10.1111/1471-0528.70250

**Published:** 2026-04-22

**Authors:** Anat Schwartz, Monica Minopoli, Chiara Di Ilio, Daniele Di Mascio, Amaranth Bhide, Basky Thilaganathan

**Affiliations:** ^1^ Fetal Medicine Unit St. George's University Hospitals NHS Foundation Trust London UK; ^2^ Department of Women's and Child Health Sciences and Public Health IRCCS A. Gemelli University Polyclinic Foundation Rome Italy; ^3^ Department of Maternal and Child Health and Urological Sciences Sapienza University of Rome Rome Italy; ^4^ Vascular Biology Research Centre, Molecular and Clinical Sciences Research Institute St. George's University of London London UK

**Keywords:** abdominal circumference, birth trauma, estimated fetal weight, prediction model, shoulder dystocia, third‐trimester ultrasound

## Abstract

**Objective:**

To develop antenatal prediction models for shoulder dystocia and birth trauma using routinely collected maternal and sonographic variables.

**Design:**

Retrospective cohort study.

**Setting:**

Single tertiary referral centre in the UK.

**Population or Sample:**

All singleton term liveborn pregnancies delivered between January 2016 and November 2024 with a third‐trimester ultrasound performed at or beyond 36 weeks' gestation.

**Methods:**

Multivariable logistic regression was used to develop antenatal prediction models for shoulder dystocia and birth trauma, incorporating maternal characteristics and fetal biometry including abdominal circumference (AC; centile or mm) and estimated fetal weight (EFW; grams or centile). Model performance was assessed using tests for multicollinearity, discrimination (area under the ROC curve, AUC) and calibration.

**Main Outcome Measures:**

Shoulder dystocia and birth trauma, the latter defined as a composite of shoulder dystocia, postpartum haemorrhage requiring blood transfusion, caesarean delivery at full dilatation, or hypoxic–ischaemic encephalopathy (HIE ≥ 1).

**Results:**

A total of 24 334 singleton term pregnancies were included; 432 (1.8%) were complicated by shoulder dystocia and 1210 (5.0%) by birth trauma. The model including maternal characteristics and AC centile demonstrated the best discrimination. For shoulder dystocia, the apparent AUC was 0.706 (95% CI 0.682–0.730); the optimism‐corrected AUC after bootstrap validation was 0.699. For birth trauma, the apparent AUC was 0.669 (95% CI 0.654–0.685); the optimism‐corrected AUC was 0.665. At a 10% false‐positive rate, sensitivity was 31.5% for shoulder dystocia and 22.8% for birth trauma, compared with 20.4% and 14.0%, respectively, using EFW ≥ 90th centile.

**Conclusions:**

Antenatal models combining fetal AC centile with maternal risk factors outperform EFW‐based thresholds currently used in clinical practice. Although discrimination was modest, the model may be useful for antenatal risk stratification and counselling, rather than as a stand‐alone clinical test. Such models may help identify pregnancies at increased risk of delivery‐related complications associated with fetal overgrowth and inform future studies evaluating targeted interventions.

## 1 Introduction

Vaginal delivery of a large‐for‐gestational‐age (LGA) neonate is a well‐established risk factor for significant maternal and neonatal morbidity secondary to obstetric or birth trauma. The most concerning acute intrapartum complication is shoulder dystocia, but LGA birth is also associated with other birth trauma such as brachial plexus injury and, in severe cases, hypoxic–ischaemic encephalopathy (HIE). For mothers, a trial of labour with an LGA fetus carries higher risks of intrapartum emergency caesarean delivery at full dilatation, obstetric anal sphincter injury (OASIS) and postpartum haemorrhage, which can have lasting consequences for maternal health and quality of life [1, 2, 3, 4, 5]. Current clinical practice typically relies on clinical evaluation or late third‐trimester ultrasound to screen for an LGA fetus. Owing to the risks associated with LGA neonates, guidelines typically recommend earlier birth based on absolute thresholds (> 4.0 or > 4.5 kg) or percentile cut‐offs (> 90th or > 95th centile) [2, 6, 7, 8]. In the absence of precise risk estimates, clinical management in pregnancies perceived to be at high risk commonly includes induction of labour from 37 weeks or elective caesarean section [4, 5, 9].

The performance of sonographic estimated fetal weight (EFW) for predicting shoulder dystocia is poor, with more than 75% cases remaining undetected [8, 10]. This reflects the fact that, although fetal weight is a significant risk factor, the majority of shoulder dystocia and related obstetric trauma occur in neonates that are not LGA. Prior studies underpinning current clinical guidelines have largely focused on the prenatal prediction of macrosomia as a proxy, rather than shoulder dystocia itself, principally because the latter is relatively infrequent and challenging to study. Recently, investigators have explored models incorporating maternal characteristics and ultrasound‐derived fetal biometry to improve antenatal risk prediction of shoulder dystocia [11]. However, predictive performance remains modest, and most approaches continue to rely on fetal size thresholds as the primary risk stratification strategy. Clinically, the key concern is not fetal size itself, but the birth trauma complications associated with increased fetal size [12, 13, 14]. Additionally, previous studies overlooked maternal characteristics such as parity, height, weight and co‐morbidities which significantly influenced birth trauma incidence, whereas there is a paucity of data on multiparametric antenatal models to predict such adverse outcomes. The aim of this study is to develop antenatal prediction models for shoulder dystocia and birth trauma using routinely collected maternal and sonographic variables.

## 2 Methods

### 2.1 Study Design and Population

We conducted a retrospective cohort study at a single tertiary referral centre (St George's University Hospitals NHS Foundation Trust, London, UK). All singleton pregnancies delivered ≥ 37 weeks between January 2016 and November 2024 with a third‐trimester ultrasound at or after 36 weeks' gestation were eligible for inclusion in the analysis. During the study period, late third‐trimester ultrasound biometry (≥ 36 weeks) was not uniformly implemented. Prior to 2020, scanning was performed at clinician discretion, with approximately 59% of term pregnancies undergoing ≥ 36‐week biometry (10 048 of ~17 000 deliveries). From 2020 onwards, routine late third‐trimester biometry was implemented in our centre. The present cohort therefore represents pregnancies with available ≥ 36‐week ultrasound assessment across both practice periods.

We excluded elective caesarean sections, intrauterine death before labour, and records with missing ultrasound or outcome data.

### 2.2 Data Sources and Management

Cases were identified through the Fetal Medicine Unit ultrasound electronic database (ViewPoint 5.6.26.148; ViewPoint Bildverarbeitung GmbH, Weßling, Germany). Maternal demographic data, obstetric history, pregnancy complications, ultrasound measurements and delivery/neonatal outcomes were extracted from hospital electronic health records. Gestational age was calculated from crown–rump length at 11–13 weeks or from the date of conception in pregnancies conceived by assisted reproductive technology. EFW was measured by experienced sonographers or obstetricians from head circumference (HC), abdominal circumference (AC) and femur length (FL) using the Hadlock‐4 formula [15]. EFW and AC centiles were derived from Intergrowth‐21 standards [16].

### 2.3 Outcomes

The primary outcomes were shoulder dystocia and birth trauma. Shoulder dystocia was defined according to the Royal College of Obstetricians and Gynaecologists (RCOG) as a vaginal cephalic birth requiring additional obstetric manoeuvres after failure of gentle traction [4]. In our electronic maternity record system, shoulder dystocia is recorded in a dedicated structured field completed by the attending midwife or obstetrician immediately following delivery. This field is specifically marked when the diagnosis is made in accordance with RCOG definitions.

Birth trauma was defined as a composite outcome including any of the following: shoulder dystocia, postpartum haemorrhage requiring blood transfusion, caesarean delivery at full dilatation, or HIE ≥ 1.

### 2.4 Statistical Analysis

Continuous variables are reported as medians (IQR) and categorical variables as counts (%). Multivariable logistic regression models were developed to predict shoulder dystocia and composite birth trauma. Candidate predictors were maternal age, ethnicity, parity, mode of conception, diabetes (gestational or pre‐gestational), induction of labour, previous vaginal delivery, previous caesarean section and sonographic measures (AC in millimetres and centiles; EFW in grams and centiles; and log‐transformed HC:AC ratio) [16]. All prespecified predictors were entered simultaneously into the multivariable models (forced‐entry approach) without automated selection procedures. Pre‐specified interaction terms were not included in the primary models in order to maintain model parsimony and minimise overfitting. Regression coefficients (*β*) and corresponding standard errors for the final multivariable models are provided in Table S2 to facilitate external validation and independent model implementation.

Separate multivariable models were constructed for each ultrasound biometric parameter to avoid collinearity between AC‐ and EFW‐derived measures. This allowed comparison of alternative biometric representations including AC centile, AC in millimetres, EFW centile and absolute EFW (grams). No univariate significance testing was used to determine variable inclusion in the prediction models. Models were fitted using complete‐case analysis for the predictors included in each model; therefore, the analytic sample size varied slightly across models.

In the primary model, maternal anthropometry was represented using body mass index (BMI), modelled per 5 kg/m^2^ increase. In sensitivity analyses, BMI was replaced with maternal height and weight (weight modelled per 5 kg) to explore alternative anthropometric parameterisations.

Continuous predictors were retained on their continuous scale. Potential non‐linearity was explored using restricted cubic splines, and predictors were retained in linear form where no evidence of non‐linearity was observed. In interval‐restricted strata (≤ 7 days and ≤ 14 days between scan and delivery), ridge penalisation was applied (rms::lrm with penalty = 1) to reduce overfitting; models in the full cohort were fitted without penalisation (penalty = 0).

Model discrimination was assessed by ROC curves with AUC and 95% CIs (DeLong).

In addition to multivariable model performance, we evaluated conventional categorical thresholds, including EFW ≥ 90th and ≥ 95th centiles, to align with thresholds used in major randomised trials. Sensitivity, specificity, positive predictive value and negative predictive value were calculated for these thresholds.

Internal validation was performed using bootstrap resampling (1000 resamples) to estimate optimism‐corrected AUC and calibration slope. Calibration was assessed using bootstrap calibration plots.

Sample size considerations were informed by recommendations for prediction model development [17]. The final analytic dataset included 432 shoulder dystocia events and 1210 composite birth trauma events.

Analyses were performed in R 4.4.3; two‐sided *p* < 0.05 was considered significant.

Patients were not involved in the design, conduct, reporting, or dissemination of this research.

## 3 Results

A total of 24 334 singleton pregnancies with scans at or beyond 36 weeks' gestation who delivered at term were included in the study. Among these, 432 cases (1.8%) were complicated by shoulder dystocia, and 1210 cases (5.0%) met criteria for birth trauma (Table 1). Of the 1210 birth trauma cases, 434 involved caesarean delivery at full dilatation, 47 were associated with HIE, and 333 required postpartum blood transfusion. On univariate analysis, several maternal and sonographic factors were associated with shoulder dystocia and birth trauma including maternal height, BMI, parity, previous obstetric history and fetal biometric parameters (Table S1). AC centile demonstrated the strongest association with both outcomes when compared with AC expressed in millimetres, EFW expressed in grams or centiles, and the log‐transformed HC:AC ratio. Across these alternative parameterisations, the AC‐centile model demonstrated the highest discriminatory performance; therefore, subsequent analyses focus on this model.

**TABLE 1:** Maternal and pregnancy characteristics by outcome group.

Characteristic	Uncomplicated birth	Shoulder dystocia	Birth trauma
Number of pregnancies	23 124	432	1210
Maternal age at exam (years)	32.0 (29.0–35.0)	32.0 (28.0–35.0)	32.0 (29.0–35.0)
Maternal BMI (kg/m^2^)	24.5 (21.9–28.3)	25.5 (22.9–29.7)	25.3 (22.5–29.0)
Maternal height (cm)	164.0 (160.0–169.0)	162.6 (158.0–168.0)	162.6 (157.5–167.6)
Non‐spontaneous conception	995 (4.3%)	14 (3.2%)	88 (7.3%)
Non‐white ethnicity	9578 (41.4%)	186 (43.1%)	521 (43.1%)
Pregestational diabetes mellitus	153 (0.7%)	9 (2.1%)	13 (1.1%)
Gestational diabetes	2848 (12.3%)	60 (13.9%)	162 (13.4%)
Nulliparous	12 298 (53.2%)	213 (49.3%)	802 (66.3%)
Previous Caesarean section	1601 (6.9%)	25 (5.8%)	116 (9.6%)
Induction of labour	10 751 (46.8%)	209 (48.4%)	642 (53.1%)
AC (centile)	59.3 (39.0–78.2)	79.7 (62.8–89.8)	72.8 (53.2–86.8)
HC (centile)	62.0 (43.2–77.9)	64.4 (47.5–79.1)	67.6 (49.6–82.2)
EFW (centile)	59.4 (39.8–76.1)	74.8 (56.9–87.0)	69.8 (51.9–83.5)
EFW (grams)	2848.0 (2654.0–3058.5)	2996.0 (2833.5–3235.2)	2944.5 (2753.2–3145.0)

*Note:* Data are presented as median (interquartile range) or *n* (%). Birth trauma is defined as the occurrence of one or more of the following outcomes: shoulder dystocia, Caesarean section at full dilatation, postpartum haemorrhage requiring blood transfusion or neonatal hypoxic–ischaemic encephalopathy. Of the 1210 birth trauma cases, 434 involved Caesarean section at full dilatation, 47 were associated with HIE, and 333 required postpartum blood transfusion.

Abbreviations: AC, abdominal circumference; BMI, body mass index; EFW, estimated fetal weight; HC, head circumference.

In multivariable antenatal models, AC centile remained the strongest predictor, and other maternal factors such as ethnicity, previous vaginal birth, diabetes and induction of labour had an independent contribution to adverse outcomes (Table 2).

**TABLE 2:** Multivariable logistic regression model for prediction of shoulder dystocia and birth trauma.

Variable	Shoulder dystocia OR (95% CI)	Composite birth trauma OR (95% CI)
AC centile (per 1‐centile increase)	1.03 (1.03–1.04)	1.02 (1.02–1.02)
Maternal age (years)	0.97 (0.96–0.99)	1.00 (0.99–1.02)
Maternal BMI (per 5 kg/m^2^ increase)	1.03 (0.95–1.13)	1.03 (0.98–1.09)
Non‐white ethnicity	1.29 (1.05–1.58)	1.32 (1.16–1.50)
Pregestational diabetes	2.16 (1.08–4.35)	1.23 (0.69–2.2)
Previous VD	1.41 (0.89–2.23)	0.74 (0.59–1.04)
Previous CS	0.89 (0.50–1.37)	1.78 (1.32–2.40)
Gestational diabetes	0.89 (0.66–1.19)	0.92 (0.77–1.10)
Nulliparous	1.16 (0.81–1.87)	1.79 (1.33–2.41)
Induction of labour	1.10 (0.90–1.35)	1.24 (1.09–1.4)

*Note:* Data are presented as odds ratios (OR) with 95% confidence intervals (CI) from logistic regression. For continuous predictors, ORs are reported per unit change as specified for each variable. All prespecified predictors were included in the multivariable model (forced‐entry approach). Birth trauma is defined as the occurrence of one or more of the following outcomes: shoulder dystocia, Caesarean section at full dilatation, postpartum haemorrhage requiring blood transfusion, or neonatal hypoxic–ischaemic encephalopathy.

Abbreviations: AC, abdominal circumference; CS, Caesarean section; VD, vaginal delivery.

**FIGURE 1:**
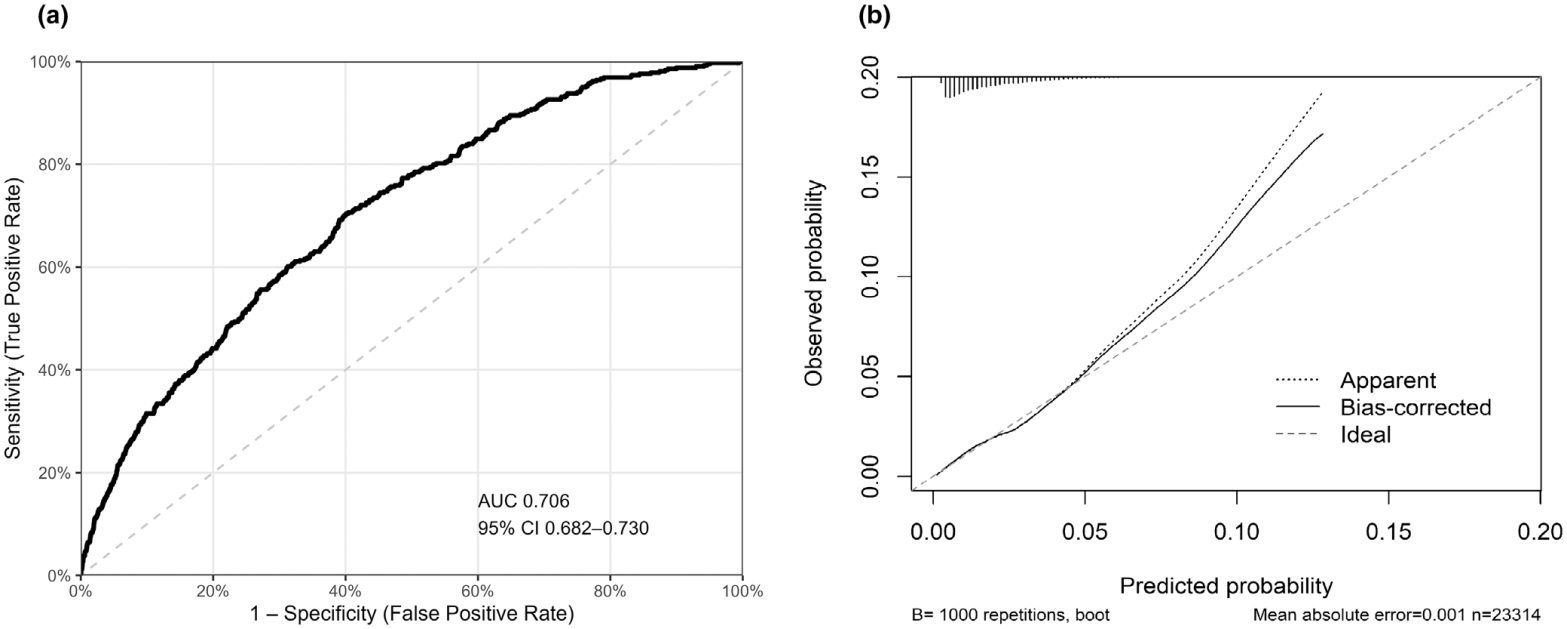
(a) Receiver‐operating characteristic (ROC) curve for prediction of shoulder dystocia using the antenatal AC‐centile multivariable model. The area under the curve (AUC) represents the apparent (in‐sample) discrimination of the model in the study cohort (AUC = 0.706, 95% CI 0.682–0.730). (b) Calibration plot for the AC‐centile multivariable model predicting shoulder dystocia. The dotted line represents the apparent calibration, the solid line the bootstrap optimism‐corrected calibration (1000 resamples), and the dashed line the ideal line of perfect calibration.

For shoulder dystocia, the apparent AUC was 0.706; the optimism‐corrected AUC after bootstrap validation was 0.699 (calibration slope 0.956), indicating moderate discrimination and showing satisfactory calibration (Figure 1). For birth trauma, the AC centile model achieved an apparent AUC of 0.669 (95% CI 0.654–0.685; Figure S1A). After bootstrap validation, the optimism‐corrected AUC was 0.665 with a calibration slope of 0.976. The adjusted associations between predictors and outcomes in the AC‐centile multivariable models are illustrated in forest plots for shoulder dystocia (Figure S2) and composite birth trauma (Figure S3). The diagnostic performance of conventional biometric thresholds and the AC‐centile multivariable models is shown in Table 3. At a 10% false positive rate (specificity 90%), the AC‐centile multivariable model achieved a sensitivity of 31.5% (CI 26.9–35.9) for shoulder dystocia and 22.8% (CI 20.4–25.5) for birth trauma, compared with 20.4% and 14.0%, respectively, when using EFW ≥ 90th centile.

**TABLE 3:** Diagnostic performance of conventional biometric thresholds and the antenatal prediction model for shoulder dystocia and birth trauma.

Clinical thresholds	Shoulder dystocia		Birth trauma	
	Sensitivity (95% CI)	Specificity (95% CI)	Sensitivity (95% CI)	Specificity (95% CI)
EFW ≥ 3.0 kg	49.5 (44.8–54.2)	68.6 (68.0–69.2)	40.8 (38.1–43.6)	68.7 (68.1–69.3)
EFW ≥ 3.5 kg	10.9 (8.3–14.2)	95.0 (94.7–95.2)	7.4 (6.1–9.1)	95.0 (94.7–95.2)
EFW ≥ 90th centile	20.4 (16.8–24.4)	92.4 (92.0–92.7)	14.0 (12.1–16.0)	92.4 (92.1–92.8)
EFW ≥ 95th centile	10.9 (8.3–14.2)	96.8 (96.5–97.0)	7.4 (6.0–9.0)	96.8 (96.6–97.1)
AC ≥ 90th centile	24.5 (20.7–28.8)	90.4 (90.1–90.8)	18.6 (16.5–20.9)	90.6 (90.3–91.0)
AC ≥ 95th centile	14.6 (11.6–18.2)	96.1 (95.8–96.3)	9.5 (8.0–11.3)	96.2 (95.9–96.4)
Prediction model (AC‐centile multivariable model)				
Sensitivity at 90 specificity	31.5 (26.9–35.9)	90.1 (90.0–90.6)	22.8 (20.4–25.5)	90.2 (90.0–90.4)
Sensitivity at 95 specificity	18.4 (14.6–22.3)	95.0 (95.0–95.4)	12.4 (10.3–14.3)	95.0 (95.0–95.3)

*Note:* Birth trauma is defined as the occurrence of one or more of the following outcomes: shoulder dystocia, Caesarean section at full dilatation, postpartum haemorrhage requiring blood transfusion or neonatal hypoxic–ischaemic encephalopathy. The AC‐centile multivariable model included AC centile and prespecified maternal covariates entered simultaneously in the multivariable model (forced‐entry approach).

Abbreviations: AC, abdominal circumference; CS, Caesarean section; EFW, estimated fetal weight; HC, head circumference; VD, vaginal delivery.

At a higher specificity threshold of 95%, sensitivities were 18.4% (CI 14.6–22.3) for shoulder dystocia and 12.4% (10.3–14.3) for birth trauma.

In comparison, EFW ≥ 95th centile achieved high specificity (96.8%) but substantially lower sensitivities of 10.9% for shoulder dystocia and 7.4% for birth trauma, indicating limited discriminatory performance at this threshold.

In secondary analyses evaluating individual components of the composite outcome, the AC‐centile multivariable model demonstrated consistent discrimination across outcomes. The optimism‐corrected AUC was 0.700 for shoulder dystocia, 0.736 for caesarean delivery at full dilatation, 0.647 for postpartum transfusion and 0.680 for hypoxic–ischaemic encephalopathy. These findings indicate that predictive performance was not driven by a single management‐dependent component but was observed across clinically relevant adverse outcomes.

## 4 Discussion

### 4.1 Main Findings

In this study of over 24 000 births, fetal AC centile demonstrated the highest discrimination for both shoulder dystocia and birth trauma compared with AC in mm, EFW in grams and EFW centile.

Combining AC centile with maternal variables such as ethnicity, previous obstetric history and diabetes in a multimodal model further improved predictive performance for shoulder dystocia and birth trauma.

However, overall discrimination remained modest, indicating that these models are more appropriately interpreted as tools for antenatal risk stratification rather than stand‐alone clinical prediction tests.

### 4.2 Strengths and Limitations

The strengths of this study include its large cohort of pregnancies with late third‐trimester ultrasound assessment and comprehensive evaluation of both demographic and sonographic predictors. By focusing on clinically relevant adverse outcomes and routinely collected variables as well as fetal size, the results have greater clinical applicability. During the early study period, late third‐trimester biometry was not universally implemented and was performed at clinician discretion. Prior to 2020, approximately 59% of term pregnancies underwent ≥ 36‐week ultrasound assessment, whereas universal screening was adopted from 2020 onwards. Accordingly, although the cohort was not strictly population‐based in the early period, a substantial proportion of pregnancies underwent late third‐trimester biometry throughout the study. Nevertheless, the model's performance may differ in settings without routine third‐trimester ultrasound assessment.

The retrospective design and single‐centre setting introduce potential biases and limit control over confounders, exposure variables and intervention bias. Additionally, some outcomes may have been under‐reported due to reliance on clinical records.

An additional methodological consideration is the potential for a treatment paradox. All women in this cohort underwent late third‐trimester ultrasound assessment, and knowledge of fetal biometry may have influenced subsequent clinical management including counselling, induction of labour, intrapartum preparedness and operative delivery decisions. Such management adaptations may attenuate the observed association between antenatal predictors and adverse outcomes, potentially leading to underestimation of the true predictive ability of fetal biometric parameters in settings where management is not modified. This effect may be particularly relevant in the post‐Boulvain era, during which clinical practice regarding suspected LGA fetuses has evolved. Accordingly, model performance may vary in healthcare settings with different management protocols, underscoring the importance of external validation.

### 4.3 Interpretation

Prior studies have demonstrated that ultrasound‐derived EFW thresholds, whether absolute (i.e., ≥ 4 kg) or as a centile (i.e., ≥ 90th centile), perform poorly for predicting shoulder dystocia and birth trauma [8, 18]. Moraitis et al. confirmed that while EFW predicts macrosomia accurately, sensitivity for shoulder dystocia is only about 20%. Similarly, Robertson et al. reported that while fetuses with EFW ≥ 95th centile are at increased risk of adverse perinatal outcomes including NICU admission, HIE and stillbirth, the absolute risk remained low with only a minority of poor outcomes attributable to shoulder dystocia. Recently, a large RCT showed that induction of labour for suspected LGA confers limited clinical benefit and does not significantly reduce shoulder dystocia compared with standard care. This highlights the limitations of macrosomia‐based management and reinforces the need for prediction models focused on clinically relevant outcomes [10].

A machine‐learning based approach has claimed a higher predictive accuracy, with a purported sensitivity of 60% for shoulder dystocia at a 25% false positive rate. However, its clinical applicability is limited as it was derived from a highly selected cohort (95% excluded due to missing data) and case‐enriched with an artificially high event rate (19%), limiting generalisability [19]. Furthermore, whereas clinical decisions regarding suspected LGA often focus on shoulder dystocia alone, our study demonstrates that a broader composite of birth trauma (5.0%) is almost three times more common than shoulder dystocia (1.8%). Importantly, this composite was intentionally broader than mechanical birth injury and was constructed to capture clinically significant maternal and neonatal consequences associated with difficult delivery in the context of suspected fetal overgrowth.

Current practice relies on categorical EFW thresholds (e.g., > 90th centile) to stratify risk [4, 10, 18]. However, sonographic estimation of fetal size is subject to random and systematic measurement errors. EFW incorporates multiple biometric parameters, and error propagation may attenuate discrimination. Furthermore, expected‐value bias has been described, whereby EFW tends to underestimate larger fetuses and overestimate smaller fetuses [20]. These methodological limitations may explain the modest performance of EFW thresholds and support evaluation of alternative biometric markers such as AC centile.

Indeed, using conventional thresholds such as EFW ≥ 90th centile, only 20% of women who experience shoulder dystocia are identified, whilst concurrently labelling approximately 1 in 12 otherwise uncomplicated pregnancies as high risk. This approach provides limited discriminatory ability and overlooks the potential value of integrating fetal biometry and maternal risk factors.

The present study demonstrates that AC centile was the most robust single predictor of adverse outcomes compared AC expressed in millimetres, EFW expressed in grams or centiles and the log‐transformed HC:AC ratio. Using an AC ≥ 90th centile model increased detection to 30% for shoulder dystocia and 25% for birth trauma, which represents a 30%–50% improvement in detection for a similar specificity to EFW‐based approaches.

As a screening tool, the AC centile model clearly outperforms EFW‐based approaches, but the AUC suggests only moderate screening performance for both shoulder dystocia and birth trauma. Whilst this limits the usefulness of these models in population screening for birth trauma, it confers many other advantages: First, it allows integration of maternal characteristics to quantify their contribution to birth trauma risk. Second, it enables estimation of birth trauma risk related to suspected LGA birth beyond shoulder dystocia alone. Third, it supports a shift from population‐based EFW thresholds towards personalised risk assessment for birth trauma. We propose that the AC centile‐based model enables targeted counselling, preparedness and informs consideration of interventions while minimising unnecessary intervention among lower‐risk women.

Previous RCTs focussed on reducing adverse outcomes related to LGA birth have relied entirely on EFW‐based thresholds as entry criteria into the study, resulting in low event rates and predominantly non‐significant clinical impacts [7, 10, 21]. The use of a prediction model including maternal and biometrical variables to enrich the eligibility criteria and inclusion of a wide spectrum of birth trauma outcomes beyond shoulder dystocia increase the feasibility of testing an intervention. Critically, external validation is required before these models are used in a clinical or research context.

## 5 Conclusions

The combination of fetal AC centile and maternal risk factors provided the highest discrimination among the antenatal models evaluated for predicting shoulder dystocia and birth trauma. Compared with conventional EFW‐based thresholds, these models demonstrated improved risk stratification, although overall predictive performance remains modest. Accordingly, the model should not be considered a stand‐alone clinical test, but rather a tool for antenatal risk stratification and research applications. Such an approach may support counselling, preparedness and future trials evaluating targeted interventions for pregnancies at increased risk of delivery‐related complications associated with fetal overgrowth.

## Author Contributions

A.S. and B.T. conceived the study and developed the study protocol. A.S. performed data curation and statistical analysis and drafted the manuscript. B.T. contributed to study design, clinical interpretation of the findings and supervised the project. M.M., A.B., D.D.M. and C.D.I. contributed to critical revision of the manuscript for important intellectual content. All authors approved the final version and agreed to be accountable for all aspects of the work.

## Funding

The authors have nothing to report.

## Ethics Statement

This study was conducted as a retrospective analysis of routinely collected clinical data. Approval was obtained from the local institutional review board of St George's University Hospitals NHS Foundation Trust. The requirement for individual informed consent was waived due to the retrospective nature of the study and use of anonymised data.

## Consent

The authors have nothing to report.

## Conflicts of Interest

The authors declare no conflicts of interest.

## Supporting information


**Figure S1:** Discriminatory and calibration performance of the antenatal AC‐centile multivariable model for prediction of composite birth trauma.
**Figure S2:** Forest plot showing adjusted odds ratios for shoulder dystocia.
**Figure S3:** Forest plot showing adjusted odds ratios for composite birth trauma.

**Table S1:** Univariate analysis of maternal and pregnancy characteristics for prediction of shoulder dystocia and birth trauma.

**Table S2:** Regression coefficients and standard errors for the multivariable antenatal prediction models.

## Data Availability

The data that support the findings of this study are available on request from the corresponding author. The data are not publicly available due to privacy or ethical restrictions.
