# Supplementary material for: Antenatal Prediction of Shoulder Dystocia and Birth Trauma Using Routine Maternal and Ultrasound Variables: Retrospective Cohort Study
Source: BJOG. 2026 Apr 22;133(11):1996–2002. doi: 10.1111/1471-0528.70250 (PMC13571882; doi:10.1111/1471-0528.70250)
Supplement: Supplementary file 1 [file BJO-133-1996-s001.docx]

**Table S2.** Regression coefficients and standard errors for the multivariable antenatal prediction models.

| **Predictor** | **β coefficient** | **Standard error** | **Adjusted OR** | **95% CI** |
| --- | --- | --- | --- | --- |
| **Shoulder dystocia model** | | | | |
| **AC centile (per 1-centile)** | 0.029 | 0.003 | 1.03 | 1.03–1.04 |
| **Maternal age (years)** | -0.030 | 0.008 | 0.97 | 0.96–0.99 |
| **Maternal BMI (per 5 kg/m²)** | 0.029 | 0.044 | 1.03 | 0.95–1.13 |
| **Non-white ethnicity** | 0.255 | 0.104 | 1.29 | 1.05–1.58 |
| **Pregestational diabetes** | 0.770 | 0.355 | 2.16 | 1.08–4.35 |
| **Previous VD** | 0.343 | 0.235 | 1.41 | 0.89–2.23 |
| **Previous CS** | -0.116 | 0.257 | 0.89 | 0.50–1.37 |
| **Gestational diabetes** | -0.116 | 0.151 | 0.89 | 0.66–1.19 |
| **Nulliparous** | 0.148 | 0.212 | 1.16 | 0.81–1.87 |
| **Induction of labour** | 0.095 | 0.103 | 1.10 | 0.90–1.35 |
| **Composite birth trauma model** | | | | |
| **AC centile (per 1-centile)** | 0.020 | 0.002 | 1.02 | 1.02–1.02 |
| **Maternal age (years)** | 0.000 | 0.007 | 1.00 | 0.99–1.02 |
| **Maternal BMI (per 5 kg/m²)** | 0.029 | 0.028 | 1.03 | 0.98–1.09 |
| **Non-white ethnicity** | 0.278 | 0.065 | 1.32 | 1.16–1.50 |
| **Pregestational diabetes** | 0.207 | 0.292 | 1.23 | 0.69–2.20 |
| **Previous VD** | -0.301 | 0.146 | 0.74 | 0.69–1.04 |
| **Previous CS** | 0.576 | 0.152 | 1.78 | 1.32–2.40 |
| **Gestational diabetes** | -0.083 | 0.091 | 0.92 | 0.77–1.10 |
| **Nulliparous** | 0.583 | 0.151 | 1.79 | 1.33–2.41 |
| **Induction of labour** | 0.215 | 0.064 | 1.24 | 1.09–1.40 |

BMI = Body Mass Index; CS = Caesarean section; VD = Vaginal delivery. Birth trauma is defined as the occurrence of one or more of the following outcomes: shoulder dystocia, Caesarean section at full dilatation, postpartum haemorrhage requiring blood transfusion, or neonatal hypoxic-ischemic encephalopathy.

β coefficients and standard errors correspond to the multivariable logistic regression models presented in Table 2.

All predictors were entered simultaneously in the multivariable models (forced-entry approach).
