# Supplementary material for: Antenatal Prediction of Shoulder Dystocia and Birth Trauma Using Routine Maternal and Ultrasound Variables: Retrospective Cohort Study
Source: BJOG. 2026 Apr 22;133(11):1996–2002. doi: 10.1111/1471-0528.70250 (PMC13571882; doi:10.1111/1471-0528.70250)
Supplement: Supplementary file 2 [file BJO-133-1996-s002.docx]

**Table S1.** Univariate analysis of maternal and pregnancy characteristics for prediction of shoulder dystocia and birth trauma. Data are presented as odds ratios (OR) with 95% confidence intervals (CI) from logistic regression. For continuous predictors, ORs are reported per unit change as specified for each variable.

| **Variable** | **Shoulder dystocia**  **OR (95% CI)** | **Birth trauma**  **OR (95% CI)** |
| --- | --- | --- |
| Maternal age (years) | 0.99 (0.97–1.00) | 1.00 (0.99–1.01) |
| Maternal BMI (per 5 kg/m² increase) | 1.160 (1.073–1.254) | 1.019 (1.017–1.022) |
| Non-white ethnicity | 1.07 (0.88–1.29) | 1.07 (0.95–1.20) |
| Non-spontaneous conception | 0.72 (0.40–1.17) | 1.74 (1.38–2.17) |
| Pregestational diabetes | 3.22 (1.52–5.98) | 1.63 (0.88–2.77) |
| Gestational diabetes | 1.15 (0.86–1.50) | 1.10 (0.93–1.30) |
| Nulliparous | 0.83 (0.69–1.01) | 1.73 (1.53–1.96) |
| Previous VD | 1.28 (1.06–1.55) | 0.51 (0.45–0.58) |
| Previous CS | 0.81 (0.52–1.18) | 1.43 (1.16–1.73) |
| Induction of labour | 1.05 (0.87–1.27) | 1.29 (1.14–1.44) |
| AC (mm) | 1.03 (1.02–1.03) | 1.02 (1.01–1.02) |
| AC centile (per 1-centile increase) | 1.03 (1.03–1.04) | 1.02 (1.02–1.02) |
| HC centile (per 1-centile increase) | 1.00 (1.00–1.01) | 1.01 (1.01–1.01) |
| EFW (per 100 g) | 1.13 (1.11–1.16) | 1.08 (1.06–1.09) |
| EFW centile (per 1-centile increase) | 1.03 (1.02–1.03) | 1.02 (1.01–1.02) |

BMI = Body Mass Index; EFW = Estimated fetal weight; AC = Abdominal circumference; HC = Head circumference; CS = Caesarean section; VD = Vaginal delivery. Birth trauma is defined as the occurrence of one or more of the following outcomes: shoulder dystocia, Caesarean section at full dilatation, postpartum haemorrhage requiring blood transfusion or neonatal hypoxic-ischemic encephalopathy.
