# Supplementary material for: Antenatal Prediction of Shoulder Dystocia and Birth Trauma Using Routine Maternal and Ultrasound Variables: Retrospective Cohort Study
Source: BJOG. 2026 Apr 22;133(11):1996–2002. doi: 10.1111/1471-0528.70250 (PMC13571882; doi:10.1111/1471-0528.70250)
Supplement: Supplementary file 3 [file BJO-133-1996-s003.zip › bjo70250-sup-0001-FigureS1-S3@CLEAN Supplementary Figure Legends 2026.03.18.docx]

**Figure legends –**

Figure 1A. Receiver-operating characteristic (ROC) curve for prediction of shoulder dystocia using the antenatal AC-centile multivariable model. The area under the curve (AUC) represents the apparent (in-sample) discrimination of the model in the study cohort (AUC = 0.706, 95% CI 0.682–0.730).

Figure 1B. Calibration plot for the AC-centile multivariable model predicting shoulder dystocia. The dotted line represents the apparent calibration, the solid line the bootstrap optimism-corrected calibration (1000 resamples), and the dashed line the ideal line of perfect calibration.

**SUPPLEMENTARY FIGURE LEGENDS**

**Figure S1.** Discriminatory and calibration performance of the antenatal AC centile multivariable model for prediction of composite birth trauma.
**(A)** Receiver‐operating characteristic (ROC) curve demonstrating the apparent discrimination of the AC centile model for prediction of composite birth trauma.
**(B)** Calibration plot comparing predicted and observed probabilities for the AC centile model, showing apparent, bootstrap bias‐corrected (B = 1000 repetitions), and ideal calibration.

**Figure S2.** Forest plot showing adjusted odds ratios with 95% confidence intervals for variables retained in the multivariable AC centile model for prediction of shoulder dystocia. OR, odds ratio; CI, confidence interval.

**Figure S3.** Forest plot showing adjusted odds ratios with 95% confidence intervals for variables retained in the multivariable AC centile model for prediction of composite birth trauma.
